# Cutaneous Adverse Effects of Elexacaftor‐Tezacaftor‐Ivacaftor: A Single Center Cohort Study on Acne Severity in Adults With Cystic Fibrosis

**DOI:** 10.1111/jocd.70528

**Published:** 2025-11-17

**Authors:** Aaron D. Smith, Catherine E. Lyons, Lindsay Somerville, R. Hal Flowers, Emily Kaplan, Dana Albon

**Affiliations:** ^1^ Department of Dermatology University of Virginia School of Medicine Charlottesville Virginia USA; ^2^ Department of Pulmonology and Critical Care Medicine University of Virginia School of Medicine Charlottesville Virginia USA

**Keywords:** acne vulgaris, acne‐QOL, CFTR modulators, cutaneous adverse effects, cystic fibrosis, drug‐induced acne, psychological, quality of life, treatment

To the Editor,

1

The advent of cystic fibrosis transmembrane conductance regulator (CFTR) modulators has revolutionized the management of cystic fibrosis (CF) [1]. Initial monotherapy with ivacaftor yielded dramatic improvements in pulmonary function for gating mutations, and dual‐combination regimens extended benefits to additional genotypes. The most recent triple‐combination therapy, elexacaftor‐tezacaftor‐ivacaftor (ETI), has achieved unprecedented gains in lung function, nutritional status, and survival in people with CF (PwCF) [2]. However, cutaneous adverse events reported in up to 24% of ETI‐treated patients may significantly undermine quality of life [2, 3, 4]. Despite its potential impact, the prevalence, timing, and patient‐reported burden of ETI‐associated acne remain poorly defined.

To address this gap, we conducted a single‐center cohort study of 31 adult PwCF (22 females, 9 males; median age, 35 years) initiating ETI (Table 1). Baseline demographics, personal and family acne history, and comorbidities were obtained via chart review and survey. Participants completed the validated Acne‐specific Quality of Life (Acne QOL) questionnaire, assessing self‐perception, role‐social, role‐emotional, and symptom‐severity domains on a 1–7 Likert scale [5]. We also recorded the anatomical distribution and timing of acne flares, along with treatments used. The project was approved by the Institutional Review Board at the University of Virginia School of Medicine.

**TABLE 1:** Summary of baseline cohort characteristics, non‐acne exacerbation, and acne exacerbation.

Variable	Non‐acne exacerbation cohort (*n* = 19)	Acne exacerbation cohort (*n* = 12)	*p*
Age, mean (SD)	35.3 (10.8)	35.0 (10.4)	NS^a^
Female, % (*n*)	68% (13/19)	75% (9/12)	NS^b^
Personal history of acne, % (*n*)	42% (8/19)	75% (9/12)	NS^b^
Family History of Acne, % (*n*)	21% (4/19)	50% (6/12)	NS^b^
Previous treatment with ivacaftor/lumacaftor, % (*n*)	73% (14/19)	83% (10/12)	NS^b^
Insulin resistance/diabetic, % (*n*)	42% (8/19)	67% (8/12)	NS^b^
[F] Use of combined oral contraceptives, % (*n*)	8% (1/13)	22% (2/9)	NS^b^
[F] Use of Depo‐Provera shots, % (*n*)	0% (0/13)	22% (2/9)	NS^b^
[F] History of polycystic ovarian syndrome, % (*n*)	8% (1/13)	0% (0/9)	NS^b^

Abbreviations: NS = No significance; SD = Standard deviation.

^a^ Welch *t*‐test.

^b^ Fisher's exact test.

Twelve patients (39%) reported subjective acne exacerbation, with a median onset of 4.5 months. Within this subgroup, facial involvement increased from 17% pre‐ETI to 100% post‐ETI (*p* < 0.001), whereas involvement of the back (from 58% to 75%) and chest (from 50% to 66%) rose without statistical significance. Mean Acne QOL scores worsened across every domain, most notably in the role‐emotional domain (mean increase from 1.5 to 2.3), but did not reach statistical significance. Nearly all affected patients used over‐the‐counter products; 57% initiated prescription topical treatments, and 50% were referred to dermatologists. No patient discontinued ETI due to acne, though several noted decreased satisfaction with therapy overall (Table 2).

**TABLE 2:** Summary of acne exacerbation cohort characteristics, pre‐ and post‐ETI exposure.

Category	Variable	Pre‐ETI	Post‐ETI	*p*
Impacted area, % (*n*)	Face	17% (2/12)	100% (12/12)	< 0.001^b^,*
	Back	58% (7/12)	75% (9/12)	NS^b^
	Chest	50% (6/12)	66% (8/12)	NS^b^
Acne QOL, mean (SD)	Self‐perception	1.56 (1.20)	2.00 (1.46)	NS^a^
	Role‐social	1.26 (1.05)	1.70 (1.37)	NS^a^
	Role‐emotional	1.57 (1.12)	2.25 (1.77)	NS^a^
	Acne symptoms	2.00 (1.32)	2.50 (1.80)	NS^a^
Treatment utilization, % (*n*)	Referral to dermatologists	33% (4/12)	50% (6/12)	NS^b^
	Over‐the‐counter products used	83% (10/12)	92% (11/12)	NS^b^
	Prescribed topical medications used	33% (4/12)	67% (8/12)	NS^b^
	Prescribed oral medications used	8% (1/12)	33% (4/12)	NS^b^
	Procedures used	0% (0/12)	17% (2/12)	NS^b^

Abbreviations: NS = No significance; SD = Standard deviation.

^a^ Welch *t*‐test.

^b^ Fisher's exact test.

* Significant (*p* < 0.05).

These findings suggest that facial ETI‐associated acne is common, temporally related to treatment initiation, and may carry a measurable psychosocial burden. Potential mechanisms are varied. CFTR‐mediated alterations in skin barrier function and shifts in cutaneous microbiota may play a role. Additionally, hormonal changes secondary to improved nutritional status could contribute. Recognizing acne as a predictable off‐target effect underscores the need for proactive dermatologic assessment and early intervention.

Standardized screening for acne should be integrated into CF clinics, with early initiation of evidence‐based therapies to prevent disease progression [2]. CF providers should be familiar with prescribing first‐line acne treatments, including benzoyl peroxide, topical retinoids, and topical antibiotics. In more severe cases, hormonal therapy or oral antibiotics may be indicated. Although previous concerns existed regarding hepatobiliary and pulmonary complications, isotretinoin can be a safe and effective option in PwCF who present with severe acne [6]. Collaboration between pulmonologists and dermatologists will streamline care pathways, ensuring that cutaneous side effects are effectively managed without compromising ETI's transformative respiratory benefits [2].

Our study is limited by its single‐center design, modest sample size, reliance on patient‐reported outcomes without objective lesion counts, and absence of standardized clinician‐graded acne severity. Future prospective multicenter investigations should quantify acne severity using standardized grading scales, explore mechanistic pathways in greater depth, and evaluate preventive and therapeutic strategies. Evidence‐based guidelines will support clinicians in balancing ETI's pulmonary advantages against its dermatologic sequelae, ensuring truly holistic care for PwCF.

## Author Contributions

A.D.S., C.E.L., L.S., and D.A. provided original conceptualization. A.D.S., L.S., E.K., and D.A. contributed to the formulation of the methodology. A.D.S. conducted a formal analysis. A.D.S. and C.E.L. wrote the manuscript, and L.S., R.H.F., E.K., and D.A. provided revisions and editing. All authors have read and approved the final manuscript.

## Disclosure

This study protocol was reviewed and approved by the University of Virginia IRB.

## Consent

Patient consent was obtained from all patients prior to their inclusion in the study and the initiation of the survey.

## Conflicts of Interest

The authors declare no conflicts of interest.

## Data Availability

The data that support the findings of this study are available from the corresponding author upon reasonable request.
